# Current and Potential Applications of Host-Defense Peptides and Proteins in Urology

**DOI:** 10.1155/2015/189016

**Published:** 2015-03-01

**Authors:** Joey Chor Yee Lo, Dirk Lange

**Affiliations:** The Stone Centre at Vancouver General Hospital, Department of Urologic Sciences, University of British Columbia, 2660 Oak Street, Vancouver, BC, Canada V6H 3Z6

## Abstract

The use of antibiotics has become increasingly disfavored as more multidrug resistant pathogens are on the rise. A promising alternative to the use of these conventional drugs includes antimicrobial peptides or host-defense peptides. These peptides typically consist of short amino acid chains with a net cationic charge and a substantial portion of hydrophobic residues. They mainly target the bacterial cell membrane but are also capable of translocating through the membrane and target intracellular components, making it difficult for bacteria to gain resistance as multiple essential cellular processes are being targeted. The use of these peptides in the field of biomedical therapies has been examined, and the different approaches to using them under various settings are constantly being discovered. In this review, we discuss the current and potential applications of these host-defense peptides in the field of urology. Besides the use of these peptides as antimicrobial agents, the value of these biological molecules has recently been expanded to their use as antitumor and anti-kidney-stone agents.

## 1. Introduction

The use of antibiotics can be dated back to the 1930s, when the sulfonamide Prontosil was introduced as the first commercially available antibiotic [1]. Several other classes of antibiotics emerged soon after, and by the 1940s, the “golden age of antibiotics” began with the introduction of penicillin [2]. In 1967, the use of antibiotics seemed so promising that the United States surgeon, William H. Stewart, even stated “…we had essentially defeated infectious diseases and could close the book on them…” [3]. However, although this statement looked true at that time, it was soon refuted when pathogenic bacteria with resistance against conventional antibiotics become increasingly prominent by the end of the 20th century; bacteria had gained resistance to multiple drugs [4]. The rise in multidrug resistant (MDR) bacteria became alarming, with a prevalence rate increase of 57% from the 1950s to the 2000s, with more resistance observed towards drugs that had been used for humans and animals for the longest time [4–6]. With MDR pathogens becoming a leading cause of nosocomial infections, and with the lack of novel, effective antibiotics, there is an urgent need to discover alternative drugs to control bacterial infections [4].

Antimicrobial peptides (AMPs) are ancient defense molecules of the innate immune system that has gained substantial attention over recent years [4, 7]. These peptides are found within a wide variety of species, including bacteria, insects, fungi, amphibians, birds, crustaceans, fishes, mammals, and humans, and can be obtained from many different sources, such as neutrophils, macrophages, and epithelial cells [7–9]. Similar to many conventional antibiotics, they have broad spectrum activity against a wide range of microorganisms, including both Gram-positive and Gram-negative bacteria, fungi, viruses, yeast, and protozoa [7, 10, 11]. However, unlike current antibiotics, these AMPs have multiple sites as targets rather than single genes or proteins [4]. Although these peptides are mainly known for their ability to disrupt the cell membrane of target organisms, they are also known for their ability to translocate through the cell membrane and alter other essential cellular activities and promote immune responses, including but not limiting to upregulating or downregulating DNA, RNA, and protein synthesis, altering gene expressions, enhancing neutrophil chemotaxis and function, promoting histamine release of mast cells, inhibiting tissue proteases, and stimulating wound healing [7, 8, 12–14]. The ability of these peptides to target multiple systems makes it difficult for bacteria to gain MDR against them, putting them at a great advantage compared to conventional antibiotics [4]. Because AMPs also stimulate the immune system in addition to being “antimicrobial,” in 2006, it was suggested that these peptides should be named “host-defense peptides” rather than “antimicrobial peptides,” where the latter name was given simply based on their initially discovered characteristic [12]. For this reason, these peptides will be referred to as “host-defense peptides” for the remaining of this review.

Host-defense peptides (HDPs) are typically 12 to 50 amino acids in length, are amphipathic with a net positive charge of +2 to +9, and consist of a substantial portion of hydrophobic residues (≥30%) [8, 12, 15]. These properties allow the peptides to interact with bacterial membranes and insert and form pores; cationic portions of the peptide interact with the negatively charged surface of the bacterial outer membrane via electrostatic bonding [16]. Hydrophobic residues of the HDPs then allow them to be inserted into the lipid bilayer and permeabilize the membrane [16]. The exact mechanisms of how HDPs go beyond the bacterial membrane and affect other essential cellular activities, however, have yet to be discovered [13].

With the promising antimicrobial effects and host immune enhancements offered by HDPs, it is not surprising that they are now of high interest in the biomedical area. Here, we discuss the current and potential applications of these peptides in the field of urology, including urinary tract infections, urological devices, urologic cancers, and kidney stone disease. A summarizing table has been included to help the reader thoroughly understand the HDPs which will be discussed throughout this review (Table 1).

## 2. Current and Potential Applications of Host-Defense Peptides in the Field of Urology

### 2.1. Urinary Tract Infections

The urinary tract functions in close proximity with fecal microflora and the outside environment [29, 30]. Yet, it must remain sterile to avoid disease [29, 30]. Our body possesses several mechanisms to help clear the urinary tract of bacteria, including urine flow, changes in urine pH, regular bladder emptying, chemical-defense components of the uroepithelium, and, when stimulated with bacteria, epithelial shedding and influx of effector immune cells [29–31]. More recently discovered is the natural prevalence of HDPs released into our urine upon stimulation with bacteria [29, 30, 32]. When our body fails to keep the urinary tract sterile, bacterial infections may take place. Urinary tract infections (UTI) are one of the most common infections in humans, affecting predominantly females of any age [17].

Several HDPs of the urinary tract have been studied to determine their expression and function, including human beta-defensin-1 (hBD-1), cathelicidin, and ribonuclease 7 [29, 30, 32]. All three peptides are amongst a group of HDPs that are highly expressed by epithelial cells of the urinary tract upon stimulation with bacteria [29, 30, 32]. Although they are present in the urine of both healthy individuals and those with UTI, their level of expression significantly increases with acute pyelonephritis or cystitis, effectively revealing antibacterial activity at micromolar concentrations [29, 30, 32].

However, despite the regular release of HDPs into our urinary tract system by uroepithelial cells, it is apparent that the level of naturally produced HDPs may sometimes not be enough, hence giving rise to the frequent occurrence of UTI. Current therapies for UTI consist of antibiotics [17]. However, as previously mentioned, the high prevalence of MDR pathogens renders the treatment ineffective. Moreover, antibiotics have been associated with adverse effects, and they are often not recommended during pregnancy or in young children [17]. To overcome this predicament, Haversen et al. have examined the effectiveness of human HDP lactoferrin and lactoferrin-derived peptides, HLD1 (EATKCFQWQRNMRKVRGPPVSCIKR-NH2) and HLD2 (TK(C)FQWQRNMRKVRGPPVS(C)IKR-NH2) in clearing UTI when administered orally [17]. Lactoferrin possesses both antimicrobial and anti-inflammatory properties and is associated with host-defense at mucosal surfaces [17]. When orally administered to female mice 30 min after instillation of 10^7^ *Escherichia coli* colony forming units (CFU) into the urinary bladder, bacterial numbers in both the kidneys and the bladder were decreased to at least 1000-fold lower than that in control groups which received either phosphate-buffered saline or water when examined 24 h after inoculation [17]. Hence, oral administration of HDPs was shown to be sufficient in treating infection and inflammation at the urinary tract, possibly via renal secretion of the peptides to the site of infection [17]; it has been previously reported that lactoferrin often leaves the body of UTI patients via the urinary tract [33]. Other studies suggest that the molecule may remain intact throughout the gastrointestinal tract, allowing it to be absorbed into the blood under certain medical conditions [34]. Although the exact mechanism of action used by lactoferrin is far from being elucidated, this finding is extremely valuable as it suggests the potential use of orally administered peptides in place of conventional antibiotics. Using this protocol, other HDPs may also be tested to access their effectiveness towards targeting other remote sites of the body when taken orally.

Alternatively, instead of introducing external sources of HDPs into our system, the expression of peptides may be upregulated as a treatment for UTI; past findings have suggested the deficiency in natural HDP production to be one of the main factors leading to the development of certain infectious diseases as well as UTI [35]. This was confirmed by a recent study, where LL-37 levels were significantly lower in UTI patients after infection compared to uninfected individuals [36]. Hence, it has been suggested that induction of certain HDPs may be an effective treatment for UTI [35]. This was confirmed by Hertting et al.; when bladder biopsies were infected with uropathogenic* E. coli*, a significant increase in cathelicidin expression was induced using vitamin D [37]. Similarly, using a mouse model, Rivas-Santiago et al. were able to upregulate the expression of *β*-defensins 3 and 4 using L-isoleucine [38]. Other approaches include the use of butyrate and vitamin D to upregulate the expression of HDPs LL-37 and cathelicidin, respectively [39, 40].

Estrogen may also indirectly induce HDPs; postmenopausal women suffer from recurrent UTI frequently as a result of low levels of estrogen, leading to structural and chemical changes in the urogenital tract which increases the likelihood of contracting UTI [41]. When estrogen is locally supplemented, the improved integrity of the urinary tract is accompanied by an increased production of HDPs, including *β*-defensins 1–3, cathelicidin, ribonuclease 7, and psoriasin [41].

Indeed certain inducers are capable of upregulating the expression of various HDPs. This is important as many studies suggest the expression of particular peptides, such as *β*-defensins 3 and 14, to serve key roles in mucosal defense of the urinary tract, combating infections associated with the system [42].

### 2.2. Medical Devices

Catheter-associated urinary tract infections (CAUTI) are one of the most common sources of healthcare-associated infections, accounting for 80% of hospital-acquired infections worldwide [43]. In the United States alone, there are approximately 450,000 cases a year, and direct treatment amounts to over $350 million annually [43, 44]. Upon insertion of the urinary catheter into the human body, bacteria adhere onto the surfaces of the implant [18, 19, 43, 45]. Once adhered, they can grow and form colonies, eventually leading to a biofilm and causing infection and encrustation [7]. Biofilms are complex, multilayered communities of microorganisms adhered onto a surface and embedded in self-produced extracellular polymeric substances, which generally consist of extracellular DNA, proteins, and polysaccharides [45–47]. The extracellular matrix reduces the diffusion of antimicrobial compounds and the close proximity of the cells facilitates horizontal gene transfer between antibiotic-resistant and nonresistant bacterial strains, making them extremely resistant to antibiotic treatment [45, 48].

Since fully developed biofilms are difficult to treat, coating urinary catheters with antimicrobial compounds prior to implantation has been of high interest to prevent the formation of biofilms [43]. To date, several different types of coatings have been tested, including antibiotics, silver, triclosan, gendine, and heparin [43]. However, these compounds are often found to be cytotoxic, are associated with the development of antibiotic resistance, or are only effective* in vitro* and not* in vivo* [19, 43].

Recently, HDPs have been examined as a potential coating for urinary catheters and ureteral stents [18, 19, 43]. Tachyplesin III (KWCFRVCYRGICYRKCR-NH2) is a HDP isolated from horseshoe crabs and has been shown to have broad spectrum activity [7]. Minardi et al. investigated the effect of coating Tachyplesin III on ureteral stents in preventing biofilm formation* in vivo* using a rat subcutaneous pouch model and found coated samples to inhibit bacterial growth up to 1000 times [7]. No drug related adverse effects were physically observed in any of the treated animals [7].

HDP implant coatings were further advanced when the use of polymer brushes was introduced [18, 19]. By covalently grafting hydrophilic copolymer (poly(N,N-dimethylacrylamide) (PDMA) and poly(N-(3-aminopropyl)methacrylamide) (PAPMA)) chains onto a surface, and conjugating them to an optimized series of HDPs, Gao et al. were able to demonstrate the effective antimicrobial activity of a peptide-brush coating [18]. Polymer brush structures served as a flexible linker between HDPs and the surface while maximizing the density of peptides per coating [18].* In vitro*, when 1–5 × 10^5^ CFU/mL of Gram-positive or Gram-negative bacteria was introduced to titanium wires (Ti-wires) coated with peptide Tet-20 (KRWRIRVRVIRKC-NH2), there was a 100,000-fold decrease in CFU for treated Ti-wires 4 hours after incubation in comparison to uncoated controls [18]. The activity was also demonstrated* in vivo* using a rat infection model; when coated and uncoated Ti-wires were implanted into subcutaneous pockets of the rat and were challenged with 10^8^ CFU of* Staphylococcus aureus* under a 7-day implantation period, CFU was decreased by 85% for treated rats compared to controls [18]. Moreover, using scanning electron microscopy (SEM), modified 50% haemolytic complement (CH50) analysis, and 3-(4,5-dimethylthiazol-2-yl)-2,5-diphenyltetrazolium bromide (MTT) assays, the authors were able to demonstrate that peptides gave insignificant platelet activation and adhesion, no complement activation in human blood, and were nontoxic to osteoblast-like cells, respectively [18]. All these results suggest HDPs to be a promising alternative to catheter coatings.

More recently, Li et al. demonstrated the effectiveness of another brush-peptide coating; they utilized allyl glycidyl ether (AGE) polymer brushes in place of PDMA/PAPMA brushes and novel peptides with salt-tolerant properties (engineered from C-terminus of salt-resistant human beta defensin 28) instead of Tet-20 [19]. These novel peptides were referred to as RK1 (RWKRWWRRKK-NH2) and RK2 (RKKRWWRRKK-NH2) [19]. The authors stated that many HDPs succumb to salt inactivation at physiological salt concentrations, and thus the HDPs must be tolerant to salt [19]. The particular brush-peptide coating was immobilized onto polydimethylsiloxane and urinary catheter surfaces and was introduced to* E. coli*,* S. aureus*, and* Candida albicans* [19].* In vitro* assays showed coated slides exhibited >70% killing activity towards all pathogens tested, with almost 100% inhibition of microbial colonization to surfaces [19]. Additionally, no toxicity towards smooth muscle cells was observed, as demonstrated using the MTT assay [19].

Indeed, brush-peptide coatings may be the next golden coating for urinary catheters to help prevent biofilm formation and infection. However, more clinically relevant* in vivo* models must be used to further test these coatings before they can be made available to the public.

### 2.3. Cancer

Besides taking a role in UTI and urologic devices, HDPs also serve a prominent role in a disease which affects 14.1 million adults per year worldwide and results in 8.2 million deaths annually: cancer [49]. In this section, we discuss the use of HDPs in bladder cancer, prostate cancer, and kidney cancer.

#### 2.3.1. Bladder Cancer

Each year, approximately 75,000 new cases of bladder cancer are diagnosed, with 20% of them leading to death [50]. If treated by transurethral resection alone, recurrence and progressiveness of nonmuscle invasive bladder cancers can occur in 80% of the cases [20]. Various chemotherapeutic drugs have been established for treatment, including postoperative adjuvant intravesicle instillations of mitomycin, epirubicin, doxorubicin, and immunotherapy with Bacillus Calmette-Guérin (BCG) [23, 51, 52]. However, current treatments have been disappointing with respect to long-term outcomes and, due to their lack of specificity, are often associated with many side effects; 38.8% of patients treated with BCG and 46.4% of patients treated with mitomycin C developed tumor recurrences within 2 years after treatment [24, 53]. BCG is also associated with moderate to severe side effects, including arthritis, febrile episodes, and risk of sepsis [23, 54, 55]. It is also common for patients to develop multidrug resistance, rendering multiple chemotherapeutics ineffective [56]. Thus, it is important to search for alternative treatments.

Interestingly, although the use of HDPs has mainly been used to target pathogens, the peptides, particularly with magainin II (GIGKFLHSAKKFGKAFVGEIMNS-NH2), have recently been reported to have significant cytotoxic effect against a wide range of cancer cell lines, including breast and lung cancers, melanoma, lymphomas, and leukemias [20–22, 57, 58]. Lehmann et al. were particularly interested in evaluating the activity of magainin II, a peptide originally isolated from the skin of African frog* Xenopus laevis*, against bladder cancer cells [20]. Using water soluble 2-(4-iodophenyl)-3-(4-nitrophenyl)-5-(2,4-disulfophenyl)-2H-tetrazolium (WST-1), bromodeoxyuridine (BrdU), and lactate dehydrogenase (LDH) assays, the authors tested antitumor activity of the HDP against 3 different bladder cell lines as well as 2 benign fibroblast cell lines as noncancerous controls [20]. Magainin II significantly inhibited both cell proliferation and DNA synthesis in all bladder cancer cells tested while having no effect on the fibroblast cell lines, demonstrating the specificity of the peptide towards cancer cell lines [20]. Potent concentrations of magainin II for tumor cells were significantly lower than that required to damage normal fibroblasts, erythrocytes, and peripheral blood lymphocytes [59]. The peptide was also shown to be highly resistant against serum proteolysis [60]. In another study using an* in vivo* severe combined immunodeficiency mouse model, introduction of magainins and their analogues to melanoma cells leads to a complete tumor regression [22]. When administered intraperitoneally to mice with ascites tumors, magainin analogues were also able to increase the rodents' survival time [21].

With magainin II looking promising, other studies looked into other HDPs that may also give similar antitumor effects [23]. One HDP family with structural and functional similarity to magainin II was the cecropin family, first isolated from the giant silk moth,* Hyalophora cecropia *[23]. Cecropins have been previously demonstrated to possess specific anticancer activity against small cell lung cancer, mammalian leukemia, gastric cancer cells, and lymphoma and colon carcinoma cell lines [61]. Using the same tests performed to evaluate the tumoricidal activity of magainin II, Lehmann and his colleagues evaluated the potency of cecropin A (KWKLFKKIEKVGQNIRDGIIKAGPAVAVVGQATQIAK-NH2) and cecropin B (KWKVFKKIEKMGRNIRNGIVKAGPAIAVLGEAKAL-NH2) against bladder cancer cells [23]. Similar to magainin II, cecropins were selective for cancer cell lines, sparing all benign cells [23]. Inhibition of cell viability and proliferation was observed at a dose-dependent manner [23]. Scanning electron microscopy allowed visualization of lethal membrane disruption in all bladder cancer cells tested, which was not present in control cells [23]. Moreover, transfection of human bladder tumor cells with cecropin genes has been shown to reduce tumor sizes in nude mouse models [62]. Cecropins have also been shown to be largely resistant against serum and urine proteolysis, giving them an advantage over classic chemotherapeutic agents such as mitomycin, which is highly unstable in urine [63].

Although both magainin and cecropins seem to be promising, some reports have suggested making synthetic modifications to further optimize their bioactivity and rate of biodegradation [24, 64]; Huang et al. worked to bypass potential proteolytic sensitivity by using nonnatural peptidomimetics [24]. They developed poly-N-substituted glycines (peptoids), which mimic the cationic, amphipathic structural feature of magainin II but consist of slight molecular changes, ensuring them to be protease-resistant and more stable [24]. Based on 3-(4,5-dimethylthiazol-2-yl)-5-(3-carboxy-methoxyphenyl)-2-(4-sulfophenyl)-2H-tetrazolium, inner salt (MTS) assays, the peptoids exhibited fast, potent cytotoxicity at low micromolar concentrations against a wide range of human cancer cell lines, with increased cytotoxicity when treatment duration was longer [24]. When subjected to primary dermal fibroblasts and red blood cells, the peptides showed minimal influence, validating their selectivity for cancer cells [24]. Looking into structure-activity correlations, hydrophobicity and amphipathicity seem to be important for the tumoricidal activity, with peptoid chains of approximately 13 residues having highest potency. The efficacy of peptoids* in vivo* has been validated using a clinically relevant orthotopic xenotransplantation model, where human breast cancer cells were implanted into immunocompromised mice [24]. When peptoid was injected 2 weeks after implantation, tumor growth was significantly inhibited [24].

Although some HDPs appear to have significant specific tumoricidal activity against cancer cell lines, the cytotoxic mechanisms remain to be discovered [23]. The mechanism behind the ability of these peptides to selectively target cancer cells while leaving benign cells spared also remains to be mapped [23]. A possibility may be due to physiochemical differences in the target cell membranes, such as differences in lipoprotein content or fluidity [23]. However, their high selectivity and tumoricidal capabilities may allow for optimal therapy* in vivo* at low therapeutic concentrations, potentially limiting any side effects associated with them [23]. It is also important to point out that the antitumor effect appears to be unaffected by the multidrug resistant cells, which is a common phenotype observed in cancer cells [23, 24]. Such advantages may allow these HDPs to be used as treatments for bladder cancer patients in the near future.

#### 2.3.2. Prostate/Kidney Cancer

Prostate cancer is the second most common cause of death in the United States, and approximately 1 of every 6 men will get diagnosed with the deadly disease during their lifetime [50]. Kidney cancer is not fatal, but with approximately 65,000 new cases a year and 20% of those resulting in death, it still presents a major concern to public health [50]. Although the two types of cancers affect different parts of the urinary tract, one of the similar characteristics between them involves the loss of human *β*-defensin-1 (DHYNCVSSGGQCLYSACPIFTKIQGTCYRGKAKCCK-NH2) [25].

Human *β*-defensin-1 (hBD-1) has been known as a HDP of the urogenital tissues for approximately two decades [32]. However, it was only within the past 10 years when the peptide started gaining extensive attention in its role as an anticancer agent [25–27, 65]. By performing immunohistochemical analysis for hBD-1 in clinical specimens of both prostate cancer and renal cell carcinoma, Donald et al. found significant cancer-specific downregulation of the peptide in 82% and 90% of prostate cancer and renal cell carcinomas, respectively, while adjacent benign regions were unaffected [25]. Based on the authors' analysis on promoter polymorphisms, it was suggested that hBD-1 acts as a tumor suppressor, promoting caspase-3-mediated apoptosis of prostate and renal cancer cells when overexpressed [26, 27]. hBD-1 is located in chromosome 8 at segment region 8p23.2, a region where multiple tumor suppressor genes reside and genetic alternations are common in prostate and renal carcinoma [26, 27].

To gain insight into how hBD-1 may affect the behavior of cancer cells, Bullard et al. cloned the peptide and expressed it ectopically in different prostate cancer cell lines, including DU145, PC3, and LNCap [27]. Introduction of the peptide showed cytotoxic effects against DU145 and PC3, but not LNCap, which suggests hBD-1 targets mainly late-stage prostate carcinoma cells [27]. As such, with the specificity against prostate and renal cancer cells and its tumor-suppressive activity, hBD-1 may be used as an effective anticancer agent [27]. It may be interesting to see what may happen when hBD-1 inducers, such as the ones mentioned in Section 2.1 of this review article, are introduced to the cancer cell lines.

### 2.4. Kidney Stone Disease

Kidney stone disease is a common pathological disorder in industrialized countries and affects 10–15% of men and 3–5% of women in the United States, with prevalence on the rise [66, 67]. The disease causes significant morbidities, and adverse effects are often experienced when the stones reach an appreciable size, which can become dislodged from the epithelial membrane [28]. Current treatments are limited to increased water intake, supervised dieting, and alkalinization agents [28, 68]. Although such treatments can provide temporary relief, they do not lower the incidence of stone formation [69].

Kidney stones can consist of different types of components, but the main component is calcium oxalate, which comprises 70% of kidney stones [70]. Two polymorphs of calcium oxalate can form, one being the monohydrate (COM) and the other being dihydrate (COD) [71]. COM is the most abundant phase in stone formers and typically constitutes the core of kidney stones [72]. They are rarely excreted via the urine by healthy individuals [70]. COD on the other hand is excreted regularly by both healthy individuals and stone formers [70]. They are less adherent and less stable and cause less damage to cell membrane compared to COM [28, 70, 73]. By preventing the dissolution of COD to COM, crystal deposition and kidney stone formation may be suppressed [73].

The inhibition of COM formation and initiation of COD polymorph has been demonstrated by osteopontin (OPN), which are highly acidic or hydrophilic peptides [70]. Although it is not consistent with the typical cationic and hydrophobic properties that define HDPs, OPN has been reported to play crucial roles as an immune modulator, being involved with chemotactic properties to recruit cells to inflammatory sites, with mediating cell activation and cytokine production and with wound healing [12, 74, 75]. As such, for this review, we consider it as a member of HDP.

OPN-derived peptides have been shown to be effective in inhibiting COM formation while promoting COD precipitation, with the main active properties being the considerable portion of aspartic acid-rich regions, motifs, phosphorylated peptides, hydrophilic residues, net negative charges, and peptide length [28, 70, 76]. Some of the most potent OPN-derived peptides have been shown to be capable of reducing COM growth by more than 90% [77]. Farmanesh et al. have recently focused on designing and screening short peptides with functional moieties made to mimic COM inhibitors [28]. Different peptide sequences were tested to determine the difference in anti-COM activity when alanine and aspartic acid amino acids were arranged differently [28]. The authors found that subtle alterations in the sequence of the acidic residues had profound effects on the anti-COM potential [28]. By using high-throughput* in situ *calcium ion-selective electrode (ISE) screening to rapidly and reproducibly screen large peptide libraries, peptide sequences were discovered which inhibited COM formation more effectively than well-known COM inhibitors, such as citrate [28]. With bulk crystallization which involved optical and scanning electron microscopy, effective inhibitors were validated and were found to have a high tendency to shift for morphology of COM crystals from hexagonal morphology to diamond-shaped platelets, possibly due to the preferential binding of peptides to particular faces of the COM [28]. Effective COM-inhibiting peptides were also found to reduce the growth rates of COM [28]. These particular results suggest these anti-COM peptides may be valuable candidates as future therapies for kidney stone formers.

**Figure 1:**
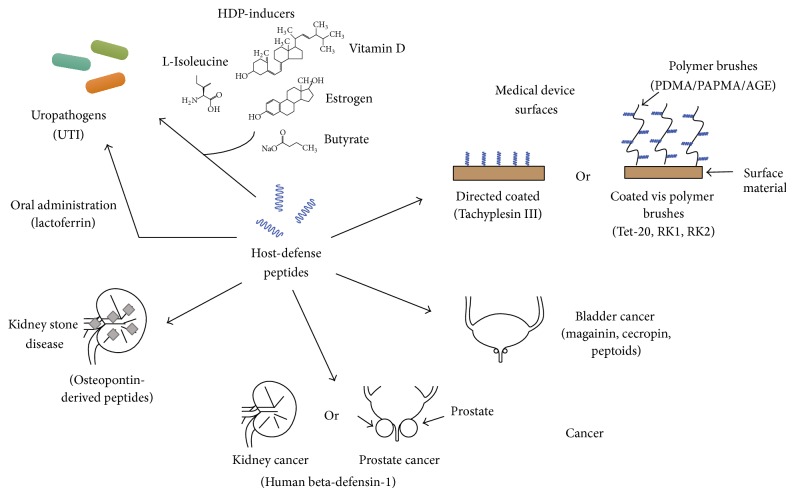
Applications of host-defense peptides in the field of urology.

Table 1 and Figure 1 have been included to aid in understanding the characteristic of HDPs discussed in this review, as well as their current and potential applications in the field of urology (Table 1, Figure 1).

## 3. Conclusions

As shown in this review, the wide-ranging functionality of HDPs against infection and disease of the urinary tract expands the peptides' activity list to well beyond the “antimicrobial peptide” originally assigned to them. From targeting bacteria and cancer cells to preventing kidney stone formation, no single peptide carried all the different traits necessary to fully treat each condition. Within urology, many potential applications of HDPs have been studied, with very promising results observed. Given that these HDPs do not appear to be susceptible to MDR bacteria and cancer cells, they may potentially form the compounds of the next “golden age” of new antimicrobials in the near future. However, before they reach that stage, further studies are required to thoroughly understand their advantages, limitations, and mechanisms of action.

## Figures and Tables

**Table 1:** Summary of host-defense peptides discussed.

Peptide name/inducers	Peptide sequence	Current/potential application in urology	References
Lactoferrin-derived peptide HLD1	EATKCFQWQRNMRKVRGPPVSCIKR-NH2	Oral administration for UTI	[17]
Lactoferrin-derived peptide HLD2	TK©FQWQRNMRKVRGPPVS©IKR-NH2		
Tachyplesin III	KWCFRVCYRGICYRKCR-NH2	Antimicrobial coating for urologic devices	[7]
Tet-20	KRWRIRVRVIRKC-NH2		[18]
RK1 (salt-tolerant)	RWKRWWRRKK		[19]
RK2 (salt-tolerant)	RKKRWWRRKK		[19]
Magainin II	GIGKFLHSAKKFGKAFVGEIMNS-NH2	Target bladder cancer cells	[20–22]
Cecropin A	KWKLFKKIEKVGQNIRDGIIKAGPAVAVVGQATQIAK-NH2		[23]
Cecropin B	KWKVFKKIEKMGRNIRNGIVKAGPAIAVLGEAKAL-NH2		
Peptoids	Most potent one analyzed: H-(Nlys-Nspe-Nspe)4-NH2		[24]
Human *β*-defensin-1	DHYNCVSSGGQCLYSACPIFTKIQGTCYRGKAKCCK-NH2		[25–27]
OPN-derived peptides	Many OPN-derived peptides were analyzed; one of the more promising ones being OPN-derived peptide D9: ADAAADDAAADAAADDAA-NH2	Target kidney stones	[28]
